# Effectiveness of Mobile App-Assisted Self-Care Interventions for Improving Patient Outcomes in Type 2 Diabetes and/or Hypertension: Systematic Review and Meta-Analysis of Randomized Controlled Trials

**DOI:** 10.2196/15779

**Published:** 2020-08-04

**Authors:** Kaifeng Liu, Zhenzhen Xie, Calvin Kalun Or

**Affiliations:** 1 Department of Industrial and Manufacturing Systems Engineering University of Hong Kong Hong Kong Hong Kong

**Keywords:** mobile app, type 2 diabetes, hypertension, self-care

## Abstract

**Background:**

Mobile app-assisted self-care interventions are emerging promising tools to support self-care of patients with chronic diseases such as type 2 diabetes and hypertension. The effectiveness of such interventions requires further exploration for more supporting evidence.

**Objective:**

A systematic review and meta-analysis of randomized controlled trials (RCTs) were conducted to examine the effectiveness of mobile app-assisted self-care interventions developed for type 2 diabetes and/or hypertension in improving patient outcomes.

**Methods:**

We followed the Cochrane Collaboration guidelines and searched MEDLINE, Cochrane Library, EMBASE, and CINAHL Plus for relevant studies published between January 2007 and January 2019. Primary outcomes included changes in hemoglobin A_1c_ (HbA_1c_) levels, systolic blood pressure (SBP), and diastolic blood pressure (DBP). Changes in other clinical-, behavioral-, knowledge-, and psychosocial-related outcomes were included as secondary outcomes. Primary outcomes and objective secondary outcomes that were reported in at least two trials were meta-analyzed; otherwise, a narrative synthesis was used for data analysis.

**Results:**

A total of 27 trials were identified and analyzed. For primary outcomes, the use of mobile app-assisted self-care interventions was associated with significant reductions in HbA_1c_ levels (standardized mean difference [SMD] −0.44, 95% CI −0.59 to −0.29; *P*<.001), SBP (SMD −0.17, 95% CI −0.31 to −0.03, *P*=.02), and DBP (SMD −0.17, 95% CI −0.30 to −0.03, *P*=.02). Subgroup analyses for primary outcomes showed that several intervention features were supportive of self-management, including blood glucose, blood pressure, and medication monitoring, communication with health care providers, automated feedback, personalized goal setting, reminders, education materials, and data visualization. In addition, 8 objective secondary outcomes were meta-analyzed, which showed that the interventions had significant lowering effects on fasting blood glucose levels and waist circumference. A total of 42 secondary outcomes were narratively synthesized, and mixed results were found.

**Conclusions:**

Mobile app-assisted self-care interventions can be effective tools for managing blood glucose and blood pressure, likely because their use facilitates remote management of health issues and data, provision of personalized self-care recommendations, patient–care provider communication, and decision making. More studies are required to further determine which combinations of intervention features are most effective in improving the control of the diseases. Moreover, evidence regarding the effects of these interventions on the behavioral, knowledge, and psychosocial outcomes of patients is still scarce, which warrants further examination.

## Introduction

Type 2 diabetes mellitus and hypertension are two common, serious medical conditions that can lead to the development of other disabling and life-threatening health problems such as stroke and heart attack. The two diseases are closely interlinked and frequently coexist. Globally, approximately 80% of type 2 diabetic patients have hypertension [1]. US statistics indicate that type 2 diabetes is 2.5 times more prevalent in hypertensive individuals than in normotensive individuals [2]. In Hong Kong, 58% of diabetic patients exhibit increased blood pressure (BP), whereas 56% of hypertensive patients have hyperglycemia [3]. These figures emphasize that the treatment and management of both of these conditions are essential.

Diabetes and hypertension management requires lifelong self-care by patients, which can be demanding and overwhelming because patients are often unskilled or unaware of self-care and also lack the necessary knowledge, tools, and support [4]. Technology is increasingly being used to help address these challenges. In particular, mobile app-assisted interventions that capitalize on smart and networking features are suggested to facilitate patient–care provider communication, information exchange, health literacy, decision making, and peer support, without the constraints of time and geography [5-11], all of which are important for self-care.

However, the effectiveness of mobile app-assisted self-care interventions developed for type 2 diabetes and/or hypertension requires more supporting evidence and thus warrants a systematic review. First, previous reviews on the use of mobile app-assisted self-care interventions for diabetes [12-15] and hypertension [16] have mainly focused on the effects of these interventions on hemoglobin A_1c_ (HbA_1c_) levels or BP and have paid relatively little attention to other variables important for effectiveness evaluation such as behavioral, knowledge, and psychosocial outcomes. Second, several randomized controlled trials (RCTs) [17-19] have recently been conducted to test the effects of such interventions on patient outcomes, and these studies must be reviewed. Third, little is known about the features of such technologies that are effective at improving blood glucose (BG) and BP management. In light of such knowledge gaps, this study systematically reviewed the existing evidence on the effectiveness of mobile app-assisted self-care interventions developed for type 2 diabetes and/or hypertension in improving patient outcomes. In this review, mobile health apps refer to mobile device-based software programs that provide health-related resources and support for the self-care of patients with type 2 diabetes and/or hypertension.

## Methods

We followed the Cochrane Collaboration guidelines for conducting this review [20]. Screening of studies for eligibility, data extraction, risk of bias assessment, and assessment of quality of evidence were performed by KL (author) and ZX (author)/MJ (researcher) independently, and any disagreement was resolved through discussion and consensus.

### Search Strategy

MEDLINE, Cochrane Library, EMBASE, and CINAHL Plus were searched for relevant studies published between January 2007 and January 2019. According to some reviews [12,14], the majority of mobile apps were released after the launch of the first generation of iPhone in 2007 and the main app stores (IOS and Android Market) in 2008; therefore, we used 2007 as the starting year in our search. The following search terms were used: (phone* or tablet*) and (monitor* or manag* or care or control) and (examin* or evaluat* or assess* or compar*) and (diabetes or diabetic* or hyperten*).

### Selection Criteria

Studies were included in the review if they (1) were RCTs, (2) examined the effects of mobile app-assisted self-care interventions relative to those of usual care on patient outcomes, (3) studied type 2 diabetic and/or hypertensive patients, and (4) were published in English-language, peer-reviewed journals. We excluded review articles, case reports, and studies that only provided an abstract.

### Study Selection

Two researchers independently read the titles and abstracts of the citations identified in the literature search, excluded clearly irrelevant studies, and reviewed the full text of the remaining articles for inclusion. The reference lists of the included studies and relevant review papers were also examined to identify missed articles.

### Data Extraction

Two researchers independently extracted the following study characteristics from each included trial: authors, publication year, study location, disease studied, sample size, HbA_1c_/BP eligibility, mean age of participants, sex ratio, trial length, features of the interventions, and changes in patient outcomes from baseline to the end of the trial in both intervention and control groups. For an RCT with multiple intervention groups relevant to this review, we split the “shared” control group into two or more groups (with smaller sample size) to apply two or more pair-wise comparisons in the meta-analysis [20]. For 2-arm cross-over RCTs, data from only the first period were extracted and analyzed.

### Outcome Measures

Primary outcomes included changes in HbA_1c_ levels, systolic BP (SBP), and diastolic BP (DBP) at the end of the trial. Changes in other outcomes, including clinical (eg, fasting BG [FBG]), behavioral (eg, medication adherence), knowledge (eg, diabetes knowledge), and psychosocial (eg, distress) outcomes, were included as secondary outcomes.

### Risk of Bias Assessment

Following the Cochrane Collaboration’s tool for risk of bias assessment [20], two researchers independently assessed the risk of bias of included trials for seven aspects: sequence generation, allocation concealment, blinding of participants and health care providers (HCPs), blinding of outcome assessors, incomplete outcome data, selective outcome, and other sources of bias. Other sources of bias included significantly different baseline characteristics between groups, presence of co-interventions, unacceptable compliance with the intervention, and different outcome assessment timings.

### Data Analysis

Primary outcomes and objective secondary outcomes were meta-analyzed when they were reported in at least two trials. We pooled data across trials using random effects models and calculated the standardized mean difference (SMD) for each outcome. The *I*^2^ statistic was calculated to measure the percentage of variation across trials due to heterogeneity, with values of 25%, 50%, and 75% indicating low, moderate, and high levels of heterogeneity, respectively [21]. The possibility of publication bias was assessed using the Egger test [22]. The meta-analysis was performed using Comprehensive Meta-Analysis version 2 (Biostat Inc) statistical software. We narratively synthesized outcomes that were reported in only one trial or were self-reported (because of the differences in the scales used across trials). In the synthesis, for each outcome we counted the numbers of trials reporting significant positive effects, no significant effects, and significant negative effects. Subgroup analyses were performed for primary outcomes when they were reported in at least two trials in each subgroup. These analyses were stratified by (1) disease type to examine the effects of the interventions in different disease populations and (2) intervention feature to identify which features are effective in glycemic and BP control.

### Assessment of Quality of Evidence

The quality of evidence for the primary and objective secondary outcomes was assessed using the Grading of Recommendations Assessment, Development and Evaluation (GRADE) system [23]. For each of the outcomes, the quality of evidence was downgraded from high by one level for each serious issue identified in the domains of risk of bias, imprecision, indirectness, inconsistency, and publication bias.

## Results

### Study Selection

**Figure 1:**
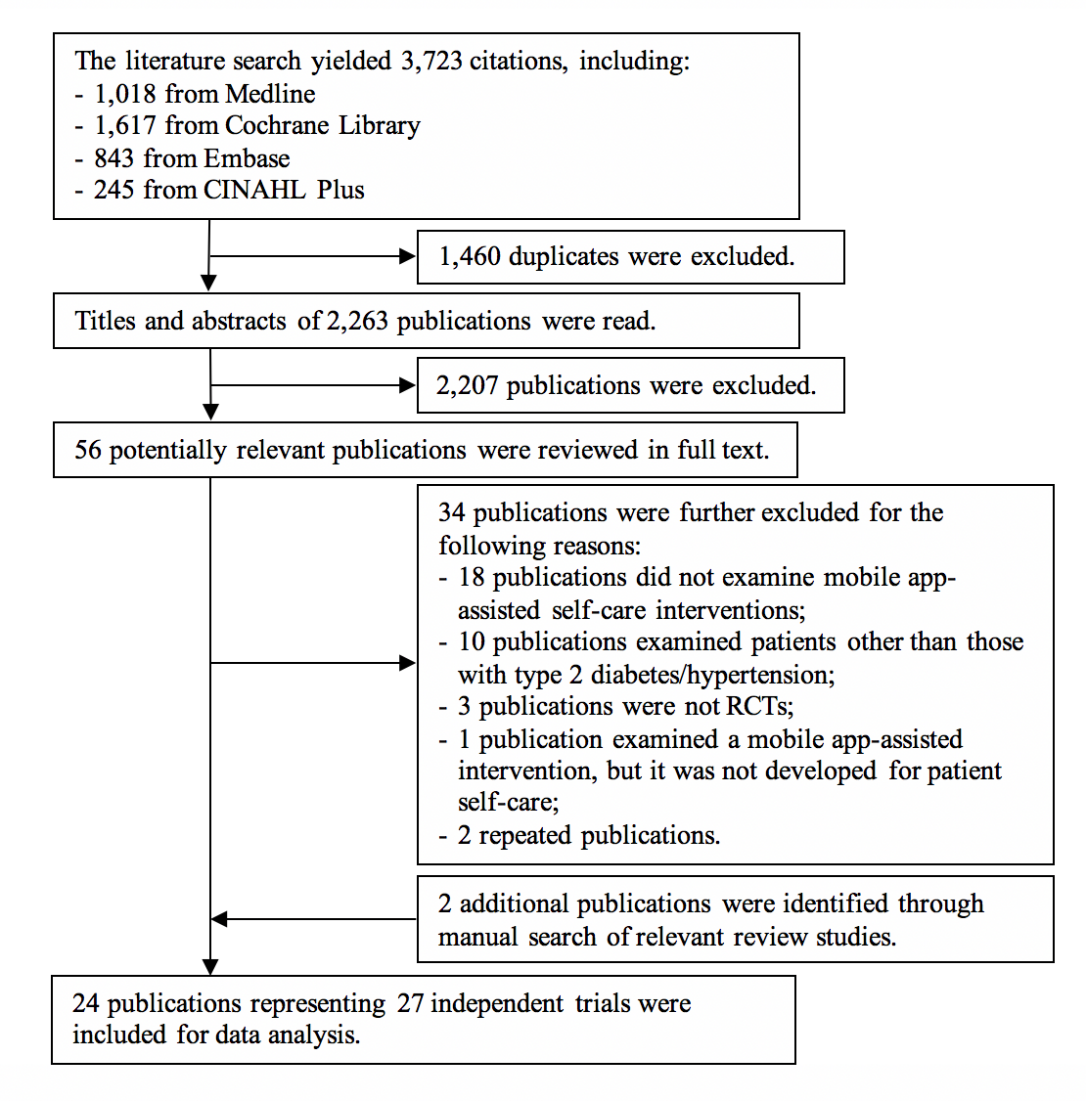
Flow diagram of the study selection process.

The study selection process (see Figure 1) identified 24 eligible publications [17-19,24-44]. Of them, the study by Holmen et al [30] had two intervention groups and the study by Quinn et al [29] had three intervention groups, all of which were relevant to this review; therefore, the control groups in these studies were split into more groups accordingly. This rendered a total of 27 independent trials for inclusion in data analysis.

### Trial Characteristics

Table 1 summarizes the characteristics of the 27 trials, and Table 2 presents the details of the trials. Fourteen features were identified in the interventions examined in the trials (see Multimedia Appendix 1 for a summary and Multimedia Appendix 2 for details). According to the taxonomies of previous studies [12,45], features were grouped into five categories: logging (ie, monitoring of BG, BP, medication, body weight, diet, physical activity, and mood), personalized feedback (ie, automated feedback, medication adjustment aid, personalized goal setting, and reminders), communication with HCPs, education materials, and data visualization.

**Table 1:** Summary of the characteristics of the 27 trials.

Characteristics		Value
**Year of publication, n (%)**		
	2007-2009	2 (7)
	2010-2012	4 (15)
	2013-2015	10 (37)
	2016-2019	11 (41)
**Study location, n (%)**		
	North America	13 (48)
	Europe	7 (26)
	Asia	5 (19)
	Africa	2 (7)
**Disease studied, n (%)**		
	Type 2 diabetes	19 (70)
	Hypertension	6 (22)
	Type 2 diabetes and/or hypertension	1 (4)
	Coexisting type 2 diabetes and hypertension	1 (4)
Sample size, median (range)		75 (14-250)
Mean age of participants in years, mean (range)		57.3 (48.4-69.5)
Proportion of male participants in %, median (range)		54 (28-76)
Trial length in months, median (range)		6 (2-12)

**Table 2:** Details of the 27 trials.

Trial, publication year, study location		Trial length	Sample	HbA_1c_^a^/BP^b^ eligibility	Intervention	Comparison treatment
**Type 2 diabetes**						
	Anzaldo-Campos et al, 2016, Mexico	10 months	IG^c^: n=102; CG^d^: n=100; mean age 52.0 years; male 38%; diabetes duration 8.3 years	HbA_1c_≥ 8%	A mobile app to facilitate self-monitoring of health-related data (eg, BG^e^ and diet) and support from clinicians, nurses, and peer educators for care management	Usual care and the provision of educational classes and health evaluation in monthly medical group visits
	Bender et al, 2017, US	6 months	IG: n=22; CG: n=23; mean age 57.6 years; male 38%; diabetes duration not reported	No limit for HbA_1c_	A mobile app for behavior tracking, a Fitbit for steps monitoring, and social media for social support and education	Usual care and a Fitbit only for daily wear
	Greenwood et al, 2015, US	6 months	IG: n=45; CG: n=45; mean age 55.7 years; male 53%; diabetes duration 8.2 years	HbA_1c_: 7.5%-10.9%	A tablet-based app and a portal to support patients’ BG monitoring and diabetes education and enable certified diabetes educators’ access to patient data for telemonitoring	Usual care, booklets and referrals for diabetes education, and evaluation of patient self-reported glucose data by certified diabetes educators
	Hansen et al, 2017, Denmark	8 months	IG: n=83; CG: n=82; mean age 58 years; male 64%; diabetes duration 12.3 years	HbA_1c_> 7.5%	A tablet-based app to enable reporting of health-related data (eg, BG and BP) and monthly communication with HCPs^f^ via video-conferencing	Usual care
	Holmen et al (1), 2014, Norway	12 months	IG: n=51; CG: n=25; mean age 57.7 years; male 64%; diabetes duration 10.6 years	HbA_1c_≥ 7.1%	A mobile phone-based system to enable vital sign monitoring, goal management, and motivational feedback	Usual care
	Holmen et al (2), 2014, Norway	12 months	IG: n=50; CG: n=25; mean age 56.9 years; male 53%; diabetes duration 9.5 years	HbA_1c_≥ 7.1%	A mobile phone-based system (to enable vital sign monitoring, goal management, and motivational feedback) and health counseling delivered by diabetes specialist nurses	Usual care
	Hsu et al, 2016, US	12 weeks	IG: n=20; CG: n=20; mean age 53.6 years; male sex not reported; diabetes duration 9.3 years	HbA_1c_: 9%-14%	A cloud-based diabetes management app supporting BG self-monitoring, insulin initiation/titration, shared decision making, and communication	Usual care with interim face-to-face visits and telephone/fax communication with educators, physicians, and/or nurses
	Karhula et al, 2015, Finland	12 months	IG: n=180; CG: n=70; mean age 66.3 years; male 56%; diabetes duration not reported	HbA_1c_> 6.5%	A mobile app for self-monitoring of health parameters (eg, BG and BP) and remote health coaching	Usual care
	Kleinman et al, 2017, India	6 months	IG: n=44; CG: n=46; mean age 48.4 years; male 70%; diabetes duration 9.2 years	HbA_1c_: 7.5%-12.5%	A smartphone app for patients and a web portal plus an app for HCPs for receiving reminders, data visualization, and providing care support to enhance self-care and collaborative care decisions	Usual care
	Nagrebetsky et al, 2013, UK	6 months	IG: n=7; CG: n=7; mean age 58 years; male 71%; diabetes duration 2.6 years	HbA_1c_: 8%-11%	A mobile phone-based telehealth platform (for self-monitoring of BG and self-titration of oral glucose-lowering medication) and monthly telephone calls (for lifestyle monitoring and change)	Usual care and monthly telephone calls for lifestyle monitoring and change
	Orsama et al, 2013, Finland	10 months	IG: n=24; CG: n=24; mean age 61.9 years; male 54%; diabetes duration not reported	HbA_1c_: 6.5%-11%; SBP^g^ >140 mm Hg or DBP^h^ >90 mm Hg	A diabetes lifestyle and self-care promotion program based on a mobile app to allow patients to report their conditions and receive system-generated feedback on health behaviors	Usual care, diabetes education, and HCP counseling
	Quinn et al, 2008, US	3 months	IG: n=13; CG: n=13; mean age 51.0 years; male 35%; diabetes duration 9.3 years	HbA_1c_ ≥ 7.5%	A mobile phone-based software to provide real-time feedback on patient BG levels, display medication instructions, incorporate hypo- and hyperglycemia treatment algorithms, and request data for diabetes management	Usual care and instructions to patients about reporting BG levels to HCPs via phone calls or fax once every 2 weeks
	Quinn et al, 2011, US (1)	12 months	IG: n=23; CG: n=19; mean age 53 years; male 52%; diabetes duration 8.3 years	HbA_1c_ ≥ 7.5%	A mobile app and patient care provider web portal to support patient self-monitoring and enable HCPs to receive health data shared by patients	Usual care
	Quinn et al, 2011, US (2)	12 months	IG: n=22; CG: n=19; mean age 53.5 years; male 46%; diabetes duration 7.8 years	HbA_1c_ ≥ 7.5%	A mobile app and patient care provider web portal to support patient self-monitoring and allow HCPs to access unanalyzed patient data	Usual care
	Quinn et al, 2011, US (3)	12 months	IG: n=62; CG: n=18; mean age 52.3 years; male 50%; diabetes duration 8.5 years	HbA_1c_ ≥ 7.5%	A mobile app and patient care provider web portal to support patient self-monitoring and allow HCPs to access analyzed patient data and evidence-based care guidelines	Usual care
	Sun et al, 2019, China	6 months	IG: n=44; CG: n=47; mean age 68.0 years; male 41%; diabetes duration 11.4 years	HbA_1c_: 7.0%-10.0%	A mobile app for self-monitoring of BG, diet, and physical activity; sharing of measurement records; and receiving HCP-provided care recommendations	Usual care
	Takenga et al, 2014, Congo	2 months	IG: n=20; CG: n=20; mean age 53.3 years; male 73%; diabetes duration not reported	No limit for HbA_1c_	A mobile system to support patients’ tracking of health conditions (eg, BG, BP, and body weight) and communication with HCPs	Usual care
	Waki et al, 2014, Japan	3 months	IG: n=27; CG: n=27; mean age 57.3 years; male 76%; diabetes duration 9.1 years	No limit for HbA_1c_	A smartphone-based system for self-monitoring of health conditions (eg, BG, BP, and diet), communication with HCPs, and receiving system’s auto-generated feedback	Usual care
	Wayne et al, 2015, Canada	6 months	IG: n=67; CG: n=64; mean age 53.2 years; male 28%; diabetes duration not reported	HbA_1c_ ≥ 7.3%	A mobile phone-supported health coach program allowing patients to track their conditions (eg, BG, diet, physical activity, and mood) and communicate with HCPs	Usual care, exercise education, and health coach support in goal setting and progress monitoring through in-person meetings/telephones
**Hypertension**						
	Kim et al, 2016, US	6 months	IG: n=52; CG: n=43; mean age 57.6 years; male 32%; hypertension duration not reported	No limit for BP	A mobile app (equipped with a BP monitoring device, electronic reminders, and a web-based disease management program for patient self-monitoring) and a reach-out program (delivered by nursing staff for education about medication, disease prevent, and chronic disease management)	Usual care and a reach-out program of the same type used in the IG
	Lakshminarayan et al, 2018, US	90 days	IG: n=34; CG: n=22; mean age 65.0 years; male 68%; hypertension duration not reported	No limit for BP	A smartphone app supporting BP self-monitoring, nurse-delivered education, and HCP-provided feedback	Usual care and education on hypertension management
	Logan et al, 2012, Canada	12 months	IG: n=55; CG: n=55; mean age 62.9 years; male 56%; hypertension duration not reported	SBP ≥130 mm Hg	A smartphone app (for BP telemonitoring and self-care) and a booklet (with information about self-management, treatments, and therapy goals)	Usual care and a booklet of the same type used in the IG
	Márquez Contreras et al, 2019, Spain	12 months	IG: n=73; CG: n=75; mean age 57.5 years; male sex 48%; hypertension duration not reported	No limit for BP	A smartphone app to promote education about hypertension and provide patients with reminders of appointments and medication	Usual care
	Moore et al, 2014, US	12 weeks	IG: n=20; CG: n=22; mean age 50.0 years; male 60%; hypertension duration not reported	BP: 140/90-180/120 mm Hg	A tablet-based app, virtual visits, instant messaging, and a nurse health coach to facilitate self-monitoring of BP and medication intake; visualization of information on actions, outcomes, and medication adjustment; and discussion about care management and goal settings	Usual care together with office visits, phone calls, and emails with HCPs for discussing care management, goal settings, and medication adjustment
	Sarfo et al, 2019, Ghana	9 months	IG: n=30; CG: n=30; mean age 55 years; male 65%; hypertension duration not reported	SBP ≥140 mm Hg	A smartphone app for monitoring and reporting of BP and medication intake, provision of motivational text messages generated based on patients’ medication adherence, and sharing of patients’ health reports with clinicians	Usual care and text messages about healthy lifestyle management and clinicians’ monthly review of patients’ BP
**Type 2 diabetes and/or hypertension**						
	Or and Tao, 2016, Hong Kong SAR, China	3 months	IG: n=33; CG: n=30; mean age 69.5 years; male 32%; diabetes duration 12.5 years; hypertension duration 10.2 years	No limit for HbA_1c_ and BP	A tablet-based self-monitoring app allowing automated recording and monitoring of BG and B*P* values and providing educational materials and decision aids	Usual care
**Type 2 diabetes and hypertension**						
	Yoo et al, 2009, Korea	3 months	IG: n=57; CG: n=54; mean age 58.2 years; male 59%; diabetes duration 6.6 years; hypertension duration 3.7 years	HbA_1c_: 6.5%-10%; BP >130/80 mm Hg	An internet-enabled, cellphone-based system coupled with a BG measuring device, an automatic BP monitor, a body weight scale, and a database providing reminders, health recommendations, and data sharing for self-care	Usual care

^a^HbA_1c_: hemoglobin A_1c_.

^b^BP: blood pressure.

^c^IG: intervention group.

^d^CG: control group.

^e^BG: blood glucose.

^f^HCP: health care provider.

^g^SBP: systolic blood pressure.

^h^DBP: diastolic blood pressure.

### Risk of Bias Assessment

**Figure 2:**
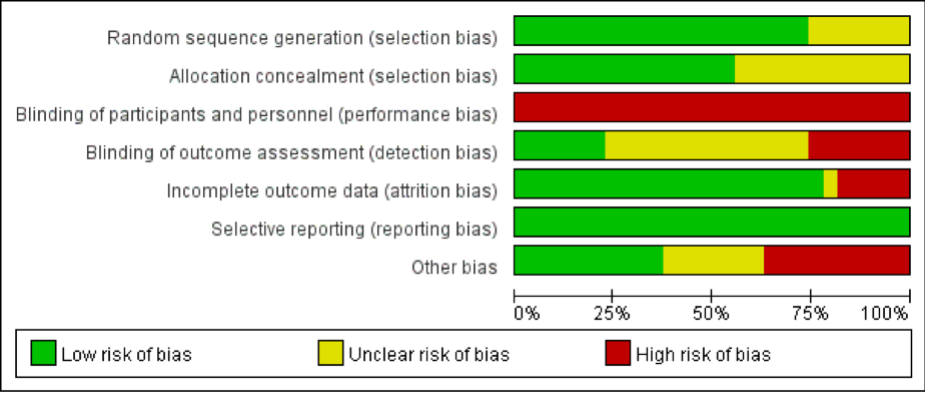
Risk of bias of the 27 trials.

**Figure 3:**
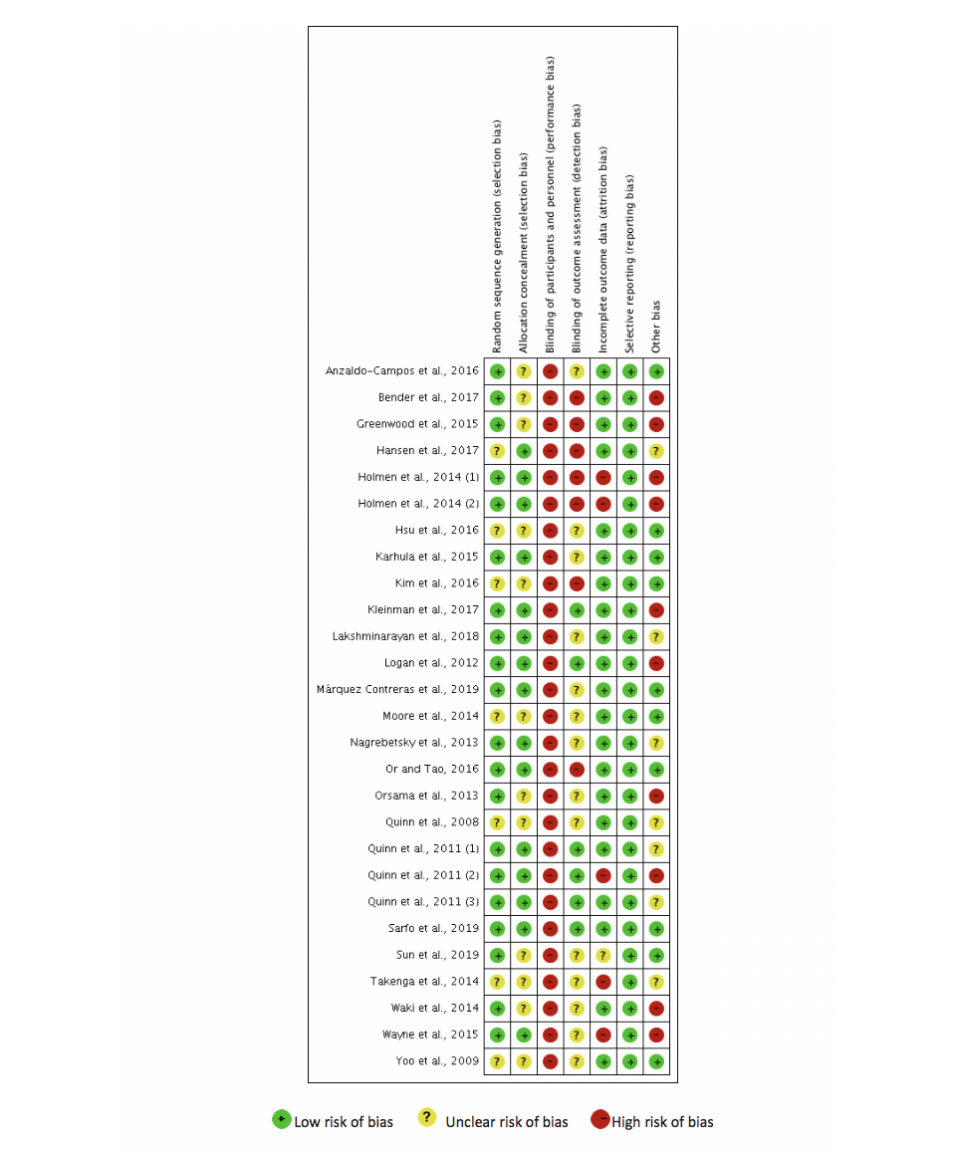
Risk of bias for each trial.

Figures 2 and 3 present the results of the risk of bias assessment.

### Meta-Analysis of the Effects on Primary Outcomes

**Figure 4:**
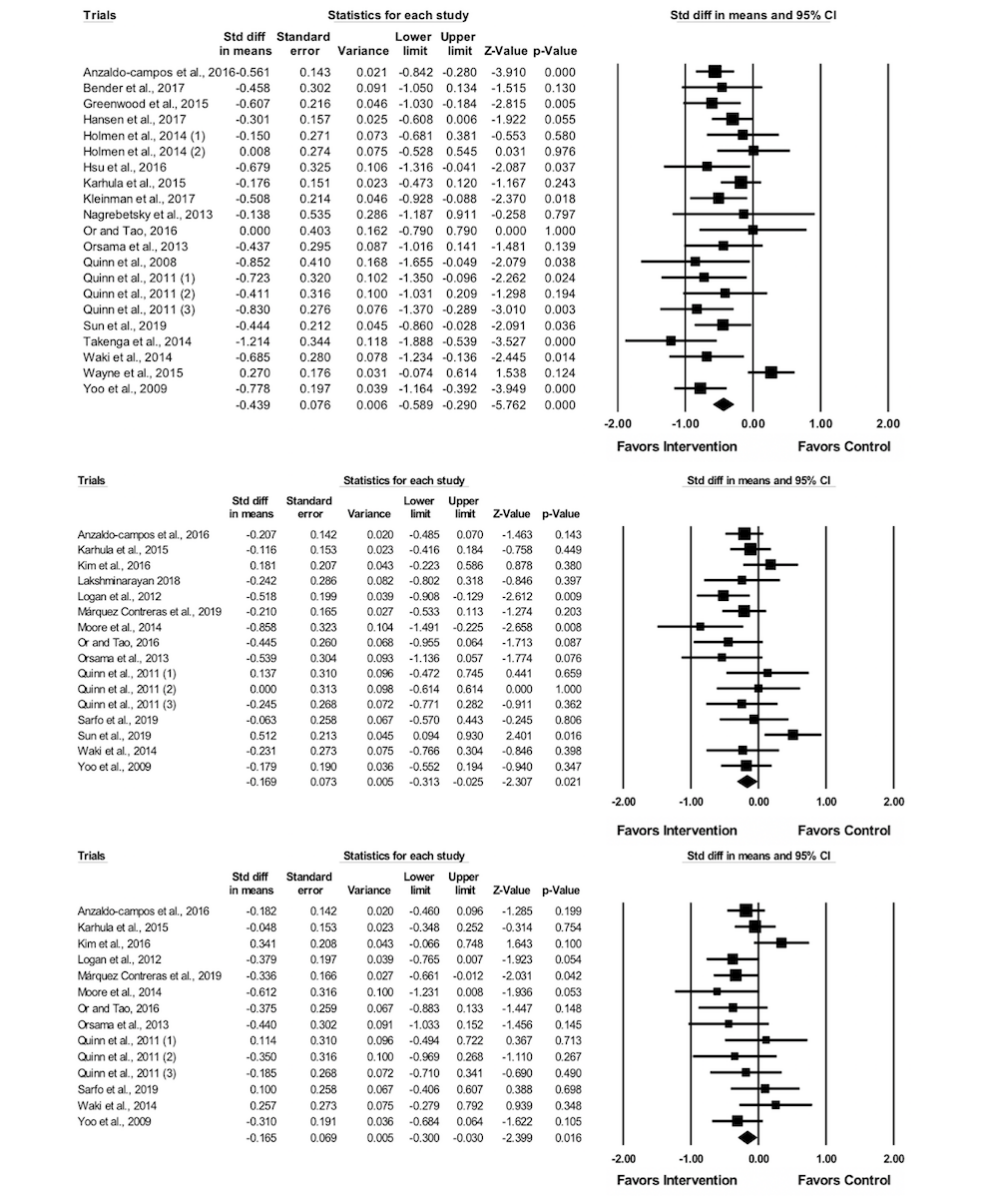
Forest plots for hemoglobin A1c (top), systolic blood pressure (middle), and diastolic blood pressure (bottom).

The meta-analysis results showed that mobile app-assisted self-care interventions were associated with significant reductions in HbA_1c_ levels (SMD −0.44, 95% CI −0.59 to −0.29, *P*<.001, corresponding to an absolute mean difference [MD] −0.49%, 95% CI −0.68 to −0.30), SBP (SMD −0.17, 95% CI −0.31 to −0.03, *P*=.02, corresponding to an absolute MD of −2.32 mm Hg, 95% CI −4.35 to −0.30), and DBP (SMD −0.17, 95% CI −0.30 to −0.03, *P*=.02, corresponding to an absolute MD of −1.53 mm Hg, 95% CI −2.78 to −0.28; Table 3). The GRADE revealed that the quality of evidence for HbA_1c_ levels, SBP, and DBP was low, moderate, and moderate, respectively (Table 3). Figure 4 presents the forest plots for the primary outcomes.

**Table 3:** Results of meta-analysis and Grading of Recommendations Assessment, Development and Evaluation assessments for hemoglobin A1c levels, systolic blood pressure, and diastolic blood pressure.

Outcomes	Trials included	Sample size	SMD^a^ (95% CI)	*P* value	*I* ^2^	Egger test		Quality of evidence (GRADE)^b^
						*t* value	*P* value	
HbA_1c_^c^ levels	21	1671	−0.44 (−0.59 to −0.29)	<.001	50	1.15	.26	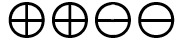 Low^d,e^
SBP^f^	16	1433	−0.17 (−0.31 to −0.03)	.02	41	0.52	.61	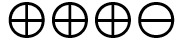 Moderate^d^
DBP^g^	14	1292	−0.17 (−0.30 to −0.03)	.02	25	0.09	.93	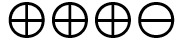 Moderate^d^

^a^SMD: standardized mean difference.

^b^GRADE: Grading of Recommendations Assessment, Development and Evaluation.

^c^HbA_1c_: hemoglobin A_1c_.

^d^Downgraded by one level for indirectness (surrogate outcome).

^e^Downgraded by one level for inconsistency (moderate heterogeneity level, *I*^2^ = 50%).

^f^SBP: systolic blood pressure.

^g^DBP: diastolic blood pressure.

### Subgroup Analysis for Primary Outcomes by Disease Type

The analysis of the HbA_1c_ outcome by disease type was not applicable because the outcome was only examined in diabetic patients and not hypertensive patients in the 27 included trials. The results of the subgroup analysis for SBP indicated that mobile app-assisted interventions led to significant reductions in SBP in hypertensive patients (SMD −0.28, 95% CI −0.51 to −0.04, *P*=.02, corresponding to an absolute MD of −4.20 mm Hg, 95% CI −7.47 to −0.93), but not in diabetic patients (SMD −0.08, 95% CI −0.29 to 0.13, *P*=.46, corresponding to an absolute MD of −0.82 mm Hg, 95% CI −3.51 to 1.87). No significant change in DBP was observed in either hypertensive patients (SMD −0.20, 95% CI −0.47 to 0.08, *P*=.17, corresponding to an absolute MD of −1.94 mm Hg, 95% CI −4.34 to 0.47) or diabetic patients (SMD −0.12, 95% CI −0.28 to 0.04, *P*=.16, corresponding to an absolute MD of −0.62 mm Hg, 95% CI −1.99 to 0.75).

### Subgroup Analysis for Primary Outcomes by Intervention Feature

Table 4 presents the results of subgroup analysis by intervention feature in relation to reductions in HbA_1c_ levels, SBP, and DBP (details see Multimedia Appendix 3, 4, and 5). The self-care interventions with the medication monitoring feature led to a significantly greater reduction in HbA_1c_ levels than those without this feature. Interventions that allowed patient-HCP communication were associated with significant reductions in HbA_1c_ while the reduction was not significant for interventions that did not have this feature, although the difference in the reduction between the subgroups (presence of the feature vs absence of the feature) was not statistically significant. As for the personalized goal-setting feature, significant reductions in HbA_1c_ levels were observed in both subgroups, but the improvement was greater when the interventions did not have this feature. For each of the other features, reductions in HbA_1c_ levels were found in both subgroups, but the difference was not significant between the subgroups.

The presence of BP monitoring, automated feedback, personalized goal setting, reminders, education materials, and data visualization features yielded significant reductions in SBP while the reductions were not significant for interventions that did not have these features, although the differences between the subgroups were not statistically significant. The presence of diet- and physical activity–monitoring features was not associated with reductions in SBP. For other features, changes in SBP were found to be similar between the subgroups.

Further, the presence of BG monitoring, automated feedback, and personalized goal-setting features was associated with reductions in DBP while the reductions were not significant for interventions that did not have these features, although the differences between the subgroups were not statistically significant. Diet monitoring, body weight monitoring, and data visualization were not associated with reductions in DBP. For other features, changes in DBP were found to be similar between the subgroups.

**Table 4:** Results of subgroup analysis by intervention feature in relation to reductions in hemoglobin A1c levels, systolic blood pressure, and diastolic blood pressure.

Features		HbA_1c_^a^ reduction	SBP^b^ reduction	DBP^c^ reduction
**Logging**				
	BG^d^	— ^e^	Δ^f^	•^g^
	BP^h^	Δ	•	Δ
	Body weight	Δ	Δ	×^i^
	Medication	•	Δ	Δ
	Diet	Δ	×	×
	Physical activity	Δ	×	Δ
	Mood	—	—	—
**Personalized feedback**				
	Automated feedback	Δ	•	•
	Medication adjustment aid	Δ	—	—
	Personalized goal setting	×	•	•
	Reminders	Δ	•	Δ
**Communication with HCP^j^**		•	—	—
**Education materials**		Δ	•	Δ
**Data visualization**		Δ	•	×

^a^HbA_1c_: hemoglobin A_1c_.

^b^SBP: systolic blood pressure.

^c^DBP: diastolic blood pressure.

^d^BG: blood glucose.

^e^—: Subgroup analysis was not performed for the feature because there were fewer than two trials in one of the subgroups.

^f^Δ: Similar changes were found between the two subgroups (presence of the feature vs absence of the feature).

^g^•: Presence of the feature was related to a more favorable effect on the outcome.

^h^BP: blood pressure.

^i^×: Absence of the feature was related to a more favorable effect on the outcome.

^j^HCP: health care provider.

### Meta-Analysis of the Effects on Objective Secondary Outcomes

A total of 8 objective secondary outcomes were meta-analyzed (Table 5). Mobile app-assisted self-care interventions had significant lowering effects on FBG (SMD −0.29, 95% CI −0.49 to −0.10, *P*=.004, corresponding to an absolute MD of −0.66 mmol/L, 95% CI −1.06 to −0.26) and waist circumference (SMD −0.23, 95% CI −0.43 to −0.04, *P*=.02, corresponding to an absolute MD of −1.62 cm, 95% CI −2.84 to −0.40), but not on body weight, BMI, total cholesterol, low-density lipoprotein (LDL) cholesterol, high-density lipoprotein (HDL) cholesterol, and triglycerides.

**Table 5:** Results of meta-analysis and Grading of Recommendations Assessment, Development and Evaluation assessments for objective secondary outcomes.

Outcomes	Trials included	Sample size	SMD^a^ (95% CI)	*P* value	*I* ^2^	Egger test		Quality of evidence (GRADE^b^)
						*t* value	*P* value	
FBG^c^	6	416	−0.29 (−0.49 to −0.10)	.004	2	2.27	.09	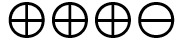 Moderate^d^
Waist circumference	4	433	−0.23 (−0.43 to −0.04)	.02	0	0.60	.61	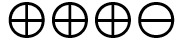 Moderate^d^
Body weight	9	682	−0.09 (−0.24 to 0.07)	.97	0	0.02	.98	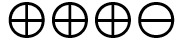 Moderate^d^
BMI	6	575	−0.06 (−0.23 to 0.12)	.53	12	3.36	.03	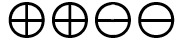 Low^d,e^
Total cholesterol	7	777	−0.18 (−0.37 to 0.02)	.07	35	0.23	.83	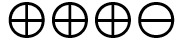 Moderate^d^
LDL^f^ cholesterol	7	734	−0.08 (−0.23 to 0.07)	.29	0	0.06	.95	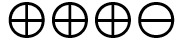 Moderate^d^
HDL^g^ cholesterol	7	743	−0.10 (−0.28 to 0.07)	.24	18	1.26	.26	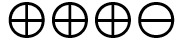 Moderate^d^
Triglycerides	7	720	−0.13 (−0.29 to 0.02)	.09	0	0.21	.84	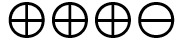 Moderate^d^

^a^SMD: standardized mean difference.

^b^GRADE: Grading of Recommendations Assessment, Development and Evaluation.

^c^FBG: fasting blood glucose.

^d^Downgraded by one level for indirectness (surrogate outcome).

^e^Downgraded by one level for publication bias.

^f^LDL: low-density lipoprotein.

^g^HDL: high-density lipoprotein.

### Narrative Synthesis of Intervention Effects

A total of 42 secondary outcomes were narratively synthesized (Table 6). The results were mixed (ie, some of the outcomes favored intervention and some other outcomes did not or for an outcome, different trials showed different results).

**Table 6:** Narrative synthesis results of the effects of mobile app-assisted self-care interventions.

Outcomes			Number of trials		
			Favoring intervention^a^	Showing no significant difference between intervention and control^b^	Favoring control^c^
**Clinical outcomes**					
	**Objectively measured**				
		Postprandial BG^d^	1 [19]		
		Right brachial-ankle pulse wave velocity		1 [40]	
		Left brachial-ankle pulse wave velocity		1 [40]	
		Adiponectin	1 [40]		
		High-sensitivity C-reactive protein		1 [40]	
		Interleukin-6		1 [40]	
		Homeostatic model assessment of insulin resistance		1 [40]	
		Waist/hip ratio		1 [44]	
		Creatinine		1 [44]	
		Medication dose			1 [35]
		Insulin dose		1 [34]	
	**Self-reported**				
		Quality of life		6 [24,28,30,32,44]	
		Diabetes symptoms		3 [29]	
**Behavioral outcomes (self-reported)**					
	**General health-related**				
		Lifestyle-/health-related activity		4 [24,30,33]	
	**Specific disease-related**				
		Adherence to medication	1 [33]	5 [17,25,27,39,42]	
		Adherence to physical activities	1 [43]	6 [25,27,30,39,41]	
		Adherence to healthy diet	1 [39]	4 [25,30,41]	
		Frequency of carbohydrate spacing	1 [25]		
		Frequency of smoking		1 [27]	
		Frequency of drinking		1 [27]	
		Frequency of communicating with physicians		1 [33]	
		Adherence to BG monitoring	2 [25,33]		
		Adherence to foot care	1 [25]		
**Knowledge outcomes (self-reported)**					
	Diabetes knowledge		1 [24]	3 [25,33,37]	
	Hypertension knowledge			3 [17,35,37]	
**Psychosocial outcomes (self-reported)**					
	**Satisfaction**				
		Satisfaction with diabetes treatment	1 [31]	1 [33]	
		Satisfaction with life		1 [28]	
	**Self-efficacy**				
		Ability to interact with health organizations and HCPs^e^		2 [30]	
		Ability to monitor the conditions and having insights into living with the conditions		2 [30]	
		Self-efficacy for medication taking/coping with diseases		4 [17,24,25,33]	
	**Emotion**				
		Emotional well-being		2 [30]	
		Positive emotion		1 [28]	
		Negative emotion		1 [28]	
		Distress		4 [29,33]	
		Depression		7 [24,28-30]	1 [34]
		Anxiety		2 [28,34]	
	**Perceived behavioral control**				
		Comfort with self-monitoring		1 [34]	
		Self-autonomous regulation		1 [17]	
		Determination about not allowing illnesses to control life		2 [30]	
		Positive and active engagement in life		2 [30]	
		Feeling of having the skills to manage disease	1 [30]	1 [30]	
		Feeling of having social support		2 [30]	

^a^Significant improvement in the outcome at the end of the trial in the intervention group compared with the control group.

^b^No significant difference in the outcome at the end of the trial between the intervention and control groups.

^c^Significant deterioration in the outcome at the end of the trial in the intervention group compared with the control group.

^d^BG: blood glucose.

^e^HCP: health care provider.

## Discussion

### Principal Findings

This systematic review identified 27 trials that examined the effectiveness of mobile app-assisted self-care interventions developed for type 2 diabetes and/or hypertension.

Overall, our review showed that the use of mobile app-assisted self-care interventions led to significant reductions in HbA_1c_ levels, SBP, and DBP—the fundamental clinical parameters in diabetic and hypertensive patients. For HbA_1c_ levels, we observed an SMD of −0.44 and an absolute MD of −0.49%. The effect size was clinically meaningful and similar to that reported in previous reviews that examined other similar types of health technology (ie, SMD −0.30 to −0.40 [15,46], absolute MD −0.40 to −0.49% [12-15,47]). As for BP, overall, the use of mobile app-assisted self-care interventions led to significant reductions in SBP (SMD −0.17, absolute MD −2.32 mm Hg) and DBP (SMD −0.17, absolute MD −1.53 mm Hg).

The subgroup analysis of BP by disease type showed that among hypertensive patients, the effect size for SBP (SMD −0.28, absolute MD −4.20 mm Hg) could be regarded as clinically important and was similar to that found in previous reviews that studied hypertensive patients (absolute MD −3.74 to −4.71 mm Hg) [48-50]. However, diabetic patients did not show significant reductions in SBP, consistent with previous reviews that examined changes in SBP among diabetic patients [15,47]. This observation could be explained by the reason that the diabetic patients examined might not have severe hypertension; therefore, room for BP reduction in those patients was relatively low. Significant reductions in DBP were not observed in either hypertensive patients or diabetic patients.

All of the reviewed interventions had more than one feature, and our subgroup analysis revealed that the effects of the features on patient outcomes varied, as follows. The presence of medication-, BG-, and BP-monitoring features were favorable in reducing HbA_1c_ levels, SBP, and DBP. Such result could be because patients already had a belief that the behaviors of monitoring of medication, BG, and BP were more immediately relevant to the control of the diseases, and, thereby, with the support of the features, patients’ engagement in the behaviors was further developed. Also, because the features could enable the tracking and organization of the health parameters in a more structured and systematic manner [16,51], patients could be more likely to be confident in their self-care and achieve improved outcomes [52]. For diet-, physical activity-, and body weight-monitoring features, their presence yielded limited efficacy. This may be due to the reason that patients might perceive the behaviors of dietary, exercise, and body weight control less directly relevant for diabetes and hypertension control, so patients’ use of the features or their engagement in the behaviors could be weak. Or even though the behaviors were considered important, it might not be easy for patients to engage in them, especially long term [53]. Education or motivational strategies may be necessary for increasing patients’ awareness about importance of diet, physical activity, and body weight control in chronic disease management. Features that enabled automated feedback and communication with HCPs were effective in improving patient outcomes. This finding could probably be explained by the fact that providing personalized feedback and suggestions based on patient health data and conditions could help patients interpret changes in their vital signs and inform them about how to deal with different situations related to the variability in their vital signs. This was especially true for patients who had low health literacy and were unable to make good use of health information. The presence of the personalized goal setting feature was favorable in reducing BP because setting specific, realistic, and timely goals could make patients more motivated to engage in planned and targeted disease management. However, this observation was not consistently reported for HbA_1c_ levels. Further evaluation is required to clarify the situations under which goal setting has a positive effect and the manner in which this feature could be used more effectively. The presence of reminder, education material, and data visualization features was associated with desirable reductions in SBP. In particular, these features could lead to higher adherence to self-care behaviors, enhanced diabetes and hypertension knowledge, and improved decision making. However, the trend was not consistently observed for HbA_1c_ levels and DBP; therefore, the efficacy of these features warrants further examination.

With respect to secondary outcomes, our meta-analyses indicated that mobile app-assisted self-care interventions had significant lowering effects on FBG and waist circumference. No significant differences were observed in body weight, BMI, total cholesterol, LDL cholesterol, HDL cholesterol, and triglycerides between the intervention and control groups, probably because the design of the interventions was less targeted for these health indexes. Our narrative synthesis indicated that in a small number of trials, the interventions were helpful in improving several clinical, behavioral, knowledge, and psychosocial outcomes. According to these trials, it appeared that such interventions have a potential to engage patients in disease management, including maintaining a healthy lifestyle, improving self-care knowledge, and addressing psychosocial needs. On the other hand, there were trials that examined these outcomes that did not show positive effects. In fact, two trials demonstrated negative effects of the mobile app-assisted self-care interventions on depression and medication dose. Given the mixed results yielded from only a small set of studies, to further understand the impacts of the interventions on these outcomes in the self-care of the diseases, more research is needed.

### Implications for Research

Our review suggests several implications for research. First, limited RCTs have emphasized behavioral, knowledge, and psychosocial aspects as primary outcomes in their examination. Further RCTs should focus more on these outcomes to obtain better understanding of whether or not, how, and to what extent mobile app-assisted health interventions change the health, self-care behaviors, and health technology adoption behaviors of patients; expand their knowledge base about health decision making and care; and influence their feelings about and attitudes toward technology-based self-care. Second, although the associations between each intervention feature and improvements in patient outcomes have been examined, information about the appropriate/optimal combinations of the features is important and limited. Future studies should further examine which combinations of intervention features are more effective for patients and disease self-care. Perhaps, the design can exploit artificial intelligence techniques to identify patients’ needs and then combine and present appropriate features tailored to those needs. In addition, our results also indicated that some features, including personalized goal setting; data visualization; and monitoring of diet, physical activity, and body weight, were not always associated with (more) improvements in patient outcomes. Further studies are required to determine the possible reasons for these observations, such as variations in patients’ acceptance and adoption of the features, engagement in the self-care activities that the features intended to support, or perceptions of the design and efficacy of the features. Design of the technology as well as patients’ attitudes toward and acceptance of the technology determine whether the technology could demonstrate its benefits and impacts [11,54-60]. Third, the implementation duration for most of the reviewed studies was 6 months or less, which is a relatively short time period for studying health behaviors related to chronic disease management. Whether the technology would motivate patients to engage in self-care activities and achieve long-term sustained benefits remains unknown.

### Implications for Practice

Our review also provides recommendations for the design and development of mobile app-assisted self-care interventions. First, our results suggest that mobile app-assisted self-care interventions should incorporate features including logging, personalized feedback, communication with HCPs, education, and data visualization in the design and implementation phases of the interventions. This suggestion is consistent with that of Greenwood et al [45] who recommended that it is important to provide a complete feedback loop between patients and their HCPs that incorporates communication, logging data, education, and personalized feedback to make the self-care process more effective. Second, some studies suggested that technical difficulties or usability problems were associated with patient withdrawals [30,32,44], whereas some other studies reported that lacking a user-friendly design is one of the most common reasons for nonadoption or low use of the technology [55,61,62]. These issues emphasize that the design and development of mobile app-assisted self-care interventions should follow human factors design methodologies and principles [55,63-66] to provide more reliable and convenient technologies for self-care. Usability tests are important in the design and development phases to identify design deficiencies. Third, some trials reported a decline in the use of such interventions over the implementation duration [34,37,41]. More effective mobile app-assisted self-care interventions should be developed to motivate patients to engage in self-care behaviors and further enhance health-related outcomes.

### Strengths and Limitations

Our review has several strengths. It provides evidence regarding the effects of mobile app-assisted self-care interventions developed for type 2 diabetes and/or hypertension on patient outcomes. In addition to HbA_1c_ levels and BP, several relevant outcomes that were scarcely examined in previous reviews are also analyzed in our review. Our review also provides an evidence-based review of the features of such interventions and their associations with improvements in glycemic and BP control. Our study has limitations. First, although type 2 diabetes and hypertension overlap in population and are closely interlinked, combining the two diseases into one systematic review may cause high heterogeneity. In this study, subgroup analysis by disease type was only conducted for primary outcomes to understand the effects of the intervention in different disease population. The effects of the intervention on the secondary outcomes should be interpreted with caution due to the variability in disease type. Second, the reported effects should be interpreted with caution because control patients in some of the reviewed trials received enhanced usual care, including additional education or phone call communications with their HCPs. Third, 42 patient outcomes were examined using narrative synthesis by simply counting their statistical significance. The effect sizes and significant levels of these outcomes were not obtained. Fourth, publication bias was detected when BMI was the examined outcome. Fifth, only English language articles were included in our review, which may have introduced language and publication bias. Finally, the review lacked an a priori and published protocol.

### Conclusions

For type 2 diabetic and/or hypertensive patients, performing self-care and maintaining a healthy lifestyle are necessary but also challenging. The use of mobile app-assisted self-care interventions appears to be effective in improving glycemic and BP management and control; however, this effectiveness was not consistent in some other outcomes. Hence, further investigations on the effects of the interventions on other outcomes are warranted. Moreover, it will be valuable to determine which combinations of features of such interventions are most effective in achieving improvements in the desired outcomes, as it can guide the optimal design of such interventions.

## Appendix

Multimedia Appendix 1 Features identified in the mobile app-assisted self-care interventions of the 27 trials.

Multimedia Appendix 2 Key features of the mobile app-assisted self-care interventions.

Multimedia Appendix 3 Effects of each intervention feature on hemoglobin A1c reduction.

Multimedia Appendix 4 Effects of each intervention feature on systolic blood pressure reduction.

Multimedia Appendix 5 Effects of each intervention feature on diastolic blood pressure reduction.

## Abbreviations

BG: blood glucose
BP: blood pressure
DBP: diastolic blood pressure
FBG: fasting blood glucose
GRADE: Grading of Recommendations Assessment, Development and Evaluation
HbA_1c_: hemoglobin A_1c_
HCP: health care provider
HDL: high-density lipoprotein
LDL: low-density lipoprotein
MD: mean difference
RCT: randomized controlled trial
SBP: systolic blood pressure
SMD: standardized mean difference
