# Correction: Effectiveness of Mobile App-Assisted Self-Care Interventions for Improving Patient Outcomes in Type 2 Diabetes and/or Hypertension: Systematic Review and Meta-Analysis of Randomized Controlled Trials

**DOI:** 10.2196/23600

**Published:** 2020-08-19

**Authors:** Kaifeng Liu, Zhenzhen Xie, Calvin Kalun Or

**Affiliations:** 1 Department of Industrial and Manufacturing Systems Engineering University of Hong Kong Hong Kong China (Hong Kong)

In “Effectiveness of Mobile App-Assisted Self-Care Interventions for Improving Patient Outcomes in Type 2 Diabetes and/or Hypertension: Systematic Review and Meta-Analysis of Randomized Controlled Trials” (JMIR mHealth 2020;8(8):e15779) the authors noted an error.

In Table 4, the symbol/indicator for “Logging–Medication” and “HbA_1c_ reduction” should be a solid dot (referring to footnote g) instead of a cross (referring to footnote i).

The correction will appear in the online version of the paper on the JMIR Publications website on August 19, 2020, together with the publication of this correction notice. Because this was made after submission to PubMed, PubMed Central, and other full-text repositories, the corrected article has also been resubmitted to those repositories.

