# Supplementary material for: Effectiveness of Mobile App-Assisted Self-Care Interventions for Improving Patient Outcomes in Type 2 Diabetes and/or Hypertension: Systematic Review and Meta-Analysis of Randomized Controlled Trials
Source: JMIR Mhealth Uhealth. 2020 Aug 4;8(8):e15779. doi: 10.2196/15779 (PMC7435643; doi:10.2196/15779)
Supplement: Multimedia Appendix 1 [file mhealth_v8i8e15779_app1.docx]

Multimedia Appendix 1. Features identified in the mobile app-assisted self-care interventions of the 27 trials.

|  | Logging | | | | | | | Personalized feedback | | | | Communication with HCPs | Education materials | Data visualization |
| --- | --- | --- | --- | --- | --- | --- | --- | --- | --- | --- | --- | --- | --- | --- |
|  | BG | BP | Body weight | Medication | Diet | Physical activity | Mood | Automated feedback | Medication adjustment aid | Personalized goal setting | Reminders |  |  |  |
| **Type 2 diabetes** | | | | | | | | | | | | | | |
| Anzaldo-Campos et al., 2016 | ✓ |  |  | ✓ | ✓ | ✓ |  |  |  |  | ✓ | ✓ | ✓ |  |
| Bender et al., 2017 |  |  | ✓ |  | ✓ | ✓ |  |  |  | ✓ |  | ✓ | ✓ |  |
| Greenwood et al., 2015 | ✓ |  |  |  |  |  |  |  |  | ✓ | ✓ | ✓ | ✓ |  |
| Hansen et al., 2017 | ✓ | ✓ | ✓ |  |  |  |  |  |  | ✓ |  | ✓ |  |  |
| Holmen et al., 2014 (1) | ✓ |  |  |  | ✓ | ✓ |  | ✓ |  | ✓ |  |  |  | ✓ |
| Holmen et al., 2014 (2) | ✓ |  |  |  | ✓ | ✓ |  | ✓ |  | ✓ |  | ✓ |  | ✓ |
| Hsu et al., 2016 | ✓ |  |  | ✓ |  |  |  |  | ✓ |  |  | ✓ |  | ✓ |
| Karhula et al., 2015 | ✓ | ✓ | ✓ |  |  |  |  |  |  | ✓ |  | ✓ |  |  |
| Kleinman et al., 2017 | ✓ |  |  |  |  |  |  | ✓ |  |  | ✓ | ✓ |  | ✓ |
| Nagrebetsky et al., 2013 | ✓ |  |  |  |  |  |  |  |  | ✓ |  | ✓ |  | ✓ |
| Orsama et al., 2013 | ✓ | ✓ | ✓ |  |  | ✓ |  | ✓ |  | ✓ |  | ✓ |  | ✓ |
| Quinn et al., 2008 | ✓ |  |  | ✓ | ✓ |  |  | ✓ | ✓ | ✓ | ✓ | ✓ | ✓ |  |
| Quinn et al., 2011 (1) | ✓ |  |  | ✓ | ✓ |  |  | ✓ |  |  |  | ✓ |  |  |
| Quinn et al., 2011 (2) | ✓ |  |  | ✓ | ✓ |  |  | ✓ |  |  |  | ✓ |  |  |
| Quinn et al., 2011 (3) | ✓ |  |  | ✓ | ✓ |  |  | ✓ |  |  |  | ✓ |  |  |
| Sun et al., 2019 | ✓ |  |  |  | ✓ | ✓ |  |  |  |  |  | ✓ |  |  |
| Takenga et al., 2014 | ✓ | ✓ | ✓ | ✓ |  | ✓ |  |  |  |  |  | ✓ |  | ✓ |
| Waki et al., 2014 | ✓ | ✓ | ✓ |  | ✓ | ✓ |  | ✓ |  |  |  | ✓ |  | ✓ |
| Wayne et al., 2015 | ✓ |  |  |  | ✓ | ✓ | ✓ |  |  | ✓ |  | ✓ |  |  |
| **Hypertension** | | | | | | | | | | | | | | |
| Kim et al., 2016 |  | ✓ |  |  |  |  |  |  |  |  | ✓ | ✓ | ✓ | ✓ |
| Lakshminarayan et al., 2018 |  | ✓ |  |  |  |  |  |  |  |  |  | ✓ |  |  |
| Logan et al., 2012 |  | ✓ |  |  |  |  |  | ✓ |  |  | ✓ | ✓ | ✓ | ✓ |
| Márquez Contreras et al., 2019 |  | ✓ |  |  |  |  |  |  |  | ✓ | ✓ | ✓ |  |  |
| Moore et al., 2014 |  | ✓ |  | ✓ |  |  |  |  | ✓ | ✓ |  | ✓ |  | ✓ |
| Sarfo et al., 2019 |  | ✓ |  | ✓ |  |  |  | ✓ |  |  |  | ✓ |  |  |
| **Type 2 diabetes and/or hypertension** | | | | | | | | | | | | | | |
| Or and Tao, 2016 | ✓ | ✓ |  |  |  |  |  | ✓ |  |  |  |  | ✓ | ✓ |
| **Type 2 diabetes and hypertension** | | | | | | | | | | | | | | |
| Yoo et al., 2009 | ✓ | ✓ | ✓ |  |  | ✓ |  | ✓ |  |  | ✓ | ✓ | ✓ |  |
