# Supplementary material for: Effectiveness of Mobile App-Assisted Self-Care Interventions for Improving Patient Outcomes in Type 2 Diabetes and/or Hypertension: Systematic Review and Meta-Analysis of Randomized Controlled Trials
Source: JMIR Mhealth Uhealth. 2020 Aug 4;8(8):e15779. doi: 10.2196/15779 (PMC7435643; doi:10.2196/15779)
Supplement: Multimedia Appendix 2 [file mhealth_v8i8e15779_app2.docx]

Multimedia Appendix 2. Key features of the mobile app-assisted self-care interventions.

| Trial | Feature |
| --- | --- |
| Anzaldo-Campos et al., 2016 | - Logging: Blood glucose (BG), medication adherence, carbohydrate intake, and physical activity - Reminders: Reminding patients to report data regarding medication adherence, carbohydrate intake, and physical activity - Communication with health care providers (HCPs): Patients shared their BG readings with HCPs; HCPs received alert messages when patients reported out-of-range BG levels or had missed appointments; and patients received visits and classes provided by a multidisciplinary care team for medical care and management. - Education materials: Video- and text-based educational materials about diabetes care |
| Bender et al., 2017 | - Logging: Daily food/calorie intake, steps, and weekly body weight - Personalized goal setting: Short- and long-term goals for weight loss - Communication with HCPs: Research staff provided personalized feedback, coaching, and support during visits. - Education materials: Facebook groups for coaching, sharing of education topics, and discussions |
| Greenwood et al., 2015 | - Logging: BG - Personalized goal setting: BG control - Reminders: Audible prompts to remind patients to measure their BG levels, use pattern management to evaluate their glucose data, and study the education materials - Communication with HCPs: Certified diabetes educators telephoned patients when self-monitoring of BG (SMBG) data indicated an urgent situation or when patients reported health or medication problems, initiated a virtual visit via asynchronous secure messaging, sent weekly summary of SMBG pattern analysis data and personalized feedback to patients and HCPs, telephoned patients regularly for discussion, and discussed medication options with patients and HCPs. - Education materials: PowerPoint slides and short video clips to deliver self-care materials |
| Hansen et al., 2017 | - Logging: BG, blood pressure (BP), and body weight - Personalized goal setting: Physicians set personalized goals for patients for HbA1c control - Communication with HCPs: Monthly video conferences with nurses and clinic-based care visits, including physician visits, every 3–6 months |
| Holmen et al., 2014 (1) | - Logging: BG, food intake, and physical activity - Automated feedback: Motivational feedback using visualization such as smiling face emoji and color codes - Personalized goal setting: The app offered a feature for personalized goal setting. - Data visualization: Visual graphs to display BG levels |
| Holmen et al., 2014 (2) | - Logging: BG, food intake, and physical activity - Automated feedback: Motivational feedback using visualization such as smiling face emoji and color codes - Personalized goal setting: The app offered a feature for personalized goal setting. - Communication with HCPs: Monthly phone-based health counseling delivered by a diabetes specialist nurse for 4 months - Data visualization: Visual graphs to display BG levels |
| Hsu et al., 2016 | - Logging: BG and medication adherence - Medication adjustment aid: Patients were provided with suggestions about medication change based on the most recent BG values and medication change protocols. - Communication with HCPs: A clinician coach’s app enabled coaches to access patient information and streamlined communication tools (secure text messages, audio, videos, and shared screen control) for communication between patients and HCPs - Data visualization: A graphical interface to display scheduled health actions about BG monitoring and measurements and medication, weekly charts to help patients and HCPs to understand the relationship between medication adherence and BG levels, and visualization for insulin titration decision support |
| Karhula et al., 2015 | - Logging: BG, BP, and body weight - Personalized goal setting: Health behavior changes - Communication with HCPs: A health coach called patients every 4–6 weeks to provide information, assistance, and support. |
| Kim et al., 2016 | - Logging: BP - Reminders: Reminders for monitoring BP - Communication with HCPs: Nursing staff had access to patients’ self-monitoring records, and a dashboard connected families, caregivers, and HCPs for monitoring and communication. - Education materials: Information about the disease condition and general health behavior recommendations - Data visualization: Graphs displaying BP trends |
| Kleinman et al., 2017 | - Logging: BG - Reminders: Reminders for BG monitoring - Communication with HCPs: Sending questions and recommendations between patients and HCPs - Automated feedback: When the BG level deviated from the normal range, the system would automatically ask follow-up questions to understand the situation and identify the problems. - Data visualization: Data visualization tools to facilitate self-care and collaborative care decisions |
| Lakshminarayan et al., 2018 | - Logging: BP - Communication with HCPs: Study investigators reviewed BP weekly, adjusted anti-hypertensive medications bi-weekly, and communicated with patients regarding medication change via phone calls/e-mail; a nurse coordinator delivered patient education. |
| Logan et al., 2012 | - Logging: BP - Automated feedback: Sending a self-care message related to hypertension control to the patients’ smartphone immediately after the recording of each BP reading; instructing patients to take additional BP measurements when the BP readings were outside the target range - Reminders: Automated voice messages were sent to patients’ home telephone requesting them to check their smartphone for a message that (1) reminded the patients to measure BP according to the schedule (when they did not adhere to their measurement schedule) and (2) instructed patients to contact their doctors immediately for advice (when their BP readings were high) - Communication with HCPs: Alerting HCPs when BP readings deviated from the normal range; encouraging patients to contact physicians when necessary - Education materials: Booklets with information on BP management - Data visualization: Graphics to display summary reports |
| Márquez Contreras et al., 2019 | - Logging: BP - Reminders: Reminders for appointments and medication intake - Personalized goal setting: BP control - Communication with HCPs: Receiving and recording physician’s advice about the prescribed treatments and posology |
| Moore et al., 2014 | - Logging: BP and medication adherence - Medication adjustment aid: Provision of medication decision support according to systolic BP and medication adherence - Personalized goal setting: Hypertension management - Communication with HCPs: HCPs helped patients to continuously progress through lifestyle change and medication adjustment using integrated instant messaging service. - Data visualization: A daily clock and weekly charts to visualize patient data, the relationship between BP control and medication adherence, and medication adjustment plan |
| Nagrebetsky et al., 2013 | - Logging: BG - Personalized goal setting: Fasting BG control - Communication with HCPs: Monitoring of BG readings by a research nursing staff via a Web-based monitoring system; encouraging the use of the telehealth system and adjustment of medications by sending standardized text messages and making monthly telephone calls to patients - Data visualization: Graphs displaying BG levels |
| Or and Tao 2016 | - Logging: BG and BP - Automated feedback: Graphical displays to show whether BG and BP values deviated from the normal ranges - Education materials: Video-based educational materials regarding diabetes and hypertension self-management - Data visualization: Graphs displaying BG and BP measurement values |
| Orsama et al., 2013 | - Logging: BG, BP, body weight, and steps - Personalized goal setting: Individual target levels for BG, BP, body weight, and steps - Automated feedback: Feedback messages based on patients’ health parameters - Communication with HCPs: Nurses studied the patients’ conditions each week and contacted patients when necessary; nurses or physicians were reminded to view patient data, contact patients, or provide feedback to patients. - Data visualization: Graphs displaying the uploaded health-related data in relation to the individual target values after each upload |
| Quinn et al., 2008 | - Logging: BG, medication adherence, and carbohydrates consumed - Automated feedback: Patients received instructions and feedback according to their self-reported vital sign values; if the reported BG was high, patients were instructed to measure their BG again or answer questions regarding the reasons for high BG levels. - Medication adjustment aid: Suggestions on medication changes - Personalized goal setting: Patient-specific target level for BG - Reminders: A guided compliance tool to direct patients to test their BG at appropriate times - Communication with HCPs: Revision of patients’ logbooks (with analyses of data, trends, and patient behaviors) and records by HCPs for communication - Education materials: E-mails to disseminate educational materials |
| Quinn et al., 2011 (1) | - Logging: BG, medication intake, and carbohydrates consumed - Automated feedback: After each upload, patients could receive real-time educational, behavioral, and motivational messages specific to the uploaded data (over 1,000 messages were incorporated into the feedback algorithm). - Communication with HCPs: A secure messaging center for patient–HCP communication; sharing of data of consenting patients with HCPs |
| Quinn et al., 2011 (2) | - Logging: BG, medication intake, and carbohydrates consumed - Automated feedback: After each upload, patients could receive real-time educational, behavioral, and motivational messages specific to the uploaded data (over 1,000 messages were incorporated into the feedback algorithm). - Communication with HCPs: A secure messaging center for patient–HCP communication; sharing of unanalyzed patient data with HCPs via a portal |
| Quinn et al., 2011 (3) | - Logging: BG, medication intake, and carbohydrates consumed - Automated feedback: After each upload, patients could receive real-time educational, behavioral, and motivational messages specific to the uploaded data (over 1,000 messages were incorporated into the feedback algorithm). - Communication with HCPs: A secure messaging center for patient–HCP communication; HCPs could access analyzed patient data, care standards, and evidence-based guidelines. |
| Sarfo et al., 2019 | - Logging: BP and medication intake - Automated feedback: Personalized motivational text messages based on patient medication adherence levels; auto-generated reports summarizing BP levels and medication adherence scores - Communication with HCPs: HCPs received patient summary reports regularly. |
| Sun et al., 2019 | - Logging: BG, diet, and physical activity - Communication with HCPs: Patients received (1) medical advice and reminders for BG monitoring via messaging app/telephone every 2 weeks sent by a medical team, (2) monthly guidance for BG monitoring and dietary advice sent by dietitian; and (3) guidance related to aerobic and resistance-based exercises |
| Takenga et al., 2014 | - Logging: BG, BP, insulin take, sports engaged in with duration, and body weight - Communication with HCPs: Physicians sent therapy plans, instructions, and recommendations using a portal to patients’ app. For emergency cases, physicians were prompted by SMS messages to check the cases and provide instructions to patients. - Data visualization: Graphs to display health-related data (e.g., BG, amount of carbohydrate consumed, units of insulin injected, and physical activity) |
| Waki et al., 2014 | - Logging: BG, BP, pedometer counts, body weight, and meals - Automated feedback: Decision-aids to show whether patients’ vital signs met the guidelines and provide personalized recommendations on lifestyle modification - Communication with HCPs: Abnormal BG and BP values were reported to physicians, and subsequent necessary interactions with patients were initiated by physicians; nutritional information of meals generated by dieticians was sent to patients - Data visualization: Graphical displays to show records of BG, BP diet, and exercise |
| Wayne et al., 2015 | - Logging: BG, exercise frequency/duration/intensity, mood, and food intake - Personalized goal setting: Health-related goals regarding BG, exercise, and diet - Communication with HCPs: Patients communicated with their health coach anytime via secure messaging, phone calls, and face-to-face meetings; health coaches accessed patient data for monitoring, offered guidelines about healthy lifestyle choices, and provided support via a Web portal when patients diverged from intended health goals and routines. |
| Yoo et al., 2009 | - Logging: BG, BP, exercise duration, and body weight - Automated feedback: Messages of encouragement, reminders, and recommendations according to the BG and BP values uploaded by patients - Reminders: Reminding patients to measure BG, BP, and body weight - Communication with HCPs: Physicians sent individualized recommendations to patients when needed. - Education materials: Text messages were used to disseminate information about diabetes, hypertension, obesity, healthy diet, and exercise. |
