# Supplementary material for: Effectiveness of Mobile App-Assisted Self-Care Interventions for Improving Patient Outcomes in Type 2 Diabetes and/or Hypertension: Systematic Review and Meta-Analysis of Randomized Controlled Trials
Source: JMIR Mhealth Uhealth. 2020 Aug 4;8(8):e15779. doi: 10.2196/15779 (PMC7435643; doi:10.2196/15779)
Supplement: Multimedia Appendix 3 [file mhealth_v8i8e15779_app3.docx]

Multimedia Appendix 3. Effects of each intervention feature on hemoglobin A1c (HbA1c) reduction.

| Features | HbA1c (%) (examined in 21 trials) | | | | | | |
| --- | --- | --- | --- | --- | --- | --- | --- |
|  | Presence of the feature | | | Absence of the feature | | | Between-group difference |
|  | n | Standardized mean difference | *P*-value | n | Standardized mean difference | *P*-value |  |
| Logging |  |  |  |  |  |  |  |
| Blood glucose^a^ |  |  |  | - |  |  |  |
| Blood pressure | 7 | −0.49 (−0.75, −0.23) | <.001 | 14 | −0.42 (−0.61, −0.23) | <.001 | .66 |
| Body weight | 7 | −0.52 (−0.76, −0.27) | <.001 | 14 | −0.40 (−0.59, −0.20) | <.001 | .45 |
| Medication | 7 | −0.68 (−0.87, −0.48) | <.001 | 14 | −0.32 (−0.50, −0.15) | <.001 | .007 |
| Diet | 11 | −0.40 (−0.64, −0.17) | .001 | 10 | −0.48 (−0.67, −0.29) | <.001 | .62 |
| Physical activity | 10 | −0.42 (−0.69, −0.16) | .002 | 11 | −0.43 (−0.57, −0.28) | <.001 | .99 |
| Mood^a^ |  |  |  | - |  |  |  |
| Personalized feedback |  |  |  |  |  |  |  |
| Automated feedback | 11 | −0.51 (−0.69, −0.33) | <.001 | 10 | −0.40 (−0.62, −0.17) | .001 | .45 |
| Medication adjustment aid | 2 | −0.75 (−1.25, −0.25) | .003 | 19 | −0.42 (−0.58, −0.26) | <.001 | .22 |
| Personalized goal setting | 10 | −0.24 (−0.44, −0.04) | .02 | 11 | −0.62 (−0.76, 0.47) | <.001 | .002 |
| Reminders | 5 | −0.62 (−0.80, −0.44) | <.001 | 16 | −0.37 (−0.55, −0.18) | <.001 | .052 |
| Communication with health care providers | 19 | −0.47 (−0.62, −0.31) | <.001 | 2 | −0.10 (−0.54, 0.34) | .65 | .13 |
| Education materials | 6 | −0.59 (−0.77, −0.41) | <.001 | 15 | −0.39 (−0.58, −0.20) | <.001 | .12 |
| Data visualization | 9 | −0.44 (−0.68, −0.19) | <.001 | 12 | −0.44 (−0.64, −0.25) | <.001 | .96 |

^a^ Subgroup analysis was not performed for the feature because there were fewer than two trials in one of the subgroups.
