# Supplementary material for: Effectiveness of Mobile App-Assisted Self-Care Interventions for Improving Patient Outcomes in Type 2 Diabetes and/or Hypertension: Systematic Review and Meta-Analysis of Randomized Controlled Trials
Source: JMIR Mhealth Uhealth. 2020 Aug 4;8(8):e15779. doi: 10.2196/15779 (PMC7435643; doi:10.2196/15779)
Supplement: Multimedia Appendix 4 [file mhealth_v8i8e15779_app4.docx]

Multimedia Appendix 4. Effects of each intervention feature on systolic blood pressure (SBP) reduction.

| Features | SBP (mmHg) (examined in 16 trials) | | | | | | |
| --- | --- | --- | --- | --- | --- | --- | --- |
|  | Presence of the feature | | | Absence of the feature | | | Between-group difference |
|  | n | Standardized mean difference | *P*-value | n | Standardized mean difference | *P*-value |  |
| Logging |  |  |  |  |  |  |  |
| Blood glucose | 10 | −0.12 (−0.30, 0.05) | .17 | 6 | −0.25 (−0.52, 0.01) | .06 | .42 |
| Blood pressure | 11 | −0.24 (−0.39, −0.10) | .001 | 5 | 0.03 (−0.28, 0.34) | .84 | .12 |
| Body weight | 4 | −0.20 (−0.40, 0.002) | .053 | 12 | −0.15 (−0.34, 0.04) | .12 | .74 |
| Medication | 6 | −0.20 (−0.42, 0.02) | .08 | 10 | −0.16 (−0.35, 0.04) | .11 | .79 |
| Diet | 6 | −0.01 (−0.28, 0.26) | .94 | 10 | −0.25 (−0.41, −0.09) | .003 | .13 |
| Physical activity | 5 | −0.11 (−0.42, 0.21) | .51 | 11 | −0.20 (−0.36, −0.04) | .01 | .60 |
| Mood^a^ |  |  |  | - |  |  |  |
| Personalized feedback |  |  |  |  |  |  |  |
| Automated feedback | 9 | −0.26 (−0.42, −0.09) | .002 | 7 | −0.10 (−0.35, 0.15) | .43 | .29 |
| Medication adjustment aid^a^ |  |  |  | - |  |  |  |
| Personalized goal setting | 4 | −0.33 (−0.61, −0.05) | .02 | 12 | −0.11 (−0.28, 0.06) | .20 | .20 |
| Reminders | 5 | −0.19 (−0.38, −0.003) | .05 | 11 | −0.16 (−0.37, 0.05) | .14 | .83 |
| Communication with health care providers^a^ |  |  |  | - |  |  |  |
| Education materials | 5 | −0.22 (−0.45, 0) | .05 | 11 | −0.14 (−0.33, 0.06) | .16 | .58 |
| Data visualization | 6 | −0.37 (−0.66, −0.07) | .02 | 10 | −0.09 (−0.23, 0.05) | .23 | .09 |

^a^ Subgroup analysis was not performed for the feature because there were fewer than two trials in one of the subgroups.
