# Supplementary material for: Effectiveness of Mobile App-Assisted Self-Care Interventions for Improving Patient Outcomes in Type 2 Diabetes and/or Hypertension: Systematic Review and Meta-Analysis of Randomized Controlled Trials
Source: JMIR Mhealth Uhealth. 2020 Aug 4;8(8):e15779. doi: 10.2196/15779 (PMC7435643; doi:10.2196/15779)
Supplement: Multimedia Appendix 5 [file mhealth_v8i8e15779_app5.docx]

Multimedia Appendix 5. Effects of each intervention feature on diastolic blood pressure (DBP) reduction.

| Features | DBP (mmHg) (examined in 14 trials) | | | | | | |
| --- | --- | --- | --- | --- | --- | --- | --- |
|  | Presence of the feature | | | Absence of the feature | | | Between-group difference |
|  | n | Standardized mean difference | *P*-value | n | Standardized mean difference | *P*-value |  |
| Logging |  |  |  |  |  |  |  |
| Blood glucose | 9 | −0.16 (−0.30, −0.02) | .02 | 5 | −0.16 (−0.49, 0.16) | .32 | .99 |
| Blood pressure | 10 | −0.17 (−0.35, 0.02) | .07 | 4 | −0.17 (−0.38, 0.05) | .13 | .99 |
| Body weight | 4 | −0.13 (−0.38, 0.12) | .31 | 10 | −0.18 (−0.35, −0.01) | .04 | .75 |
| Medication | 6 | −0.17 (−0.36, 0.02) | .08 | 8 | −0.16 (−0.36, 0.04) | .11 | .95 |
| Diet | 5 | −0.11 (−0.31, 0.09) | .29 | 9 | −0.20 (−0.39, −0.02) | .03 | .50 |
| Physical activity | 4 | −0.18 (−0.41, 0.04) | .12 | 10 | −0.16 (−0.33, 0.02) | .08 | .87 |
| Mood^a^ |  |  |  | - |  |  |  |
| Personalized feedback |  |  |  |  |  |  |  |
| Automated feedback | 9 | −0.20 (−0.37, −0.04) | .02 | 5 | −0.14 (−0.38, 0.11) | .28 | .67 |
| Medication adjustment aid^a^ |  |  |  | - |  |  |  |
| Personalized goal setting | 4 | −0.27 (−0.50, −0.04) | .02 | 10 | −0.11 (−0.28, 0.06) | .19 | .28 |
| Reminders | 5 | −0.19 (−0.41, 0.05) | .12 | 9 | −0.14 (−0.31, 0.04) | .13 | .73 |
| Communication with health care providers^a^ |  |  |  | - |  |  |  |
| Education materials | 5 | −0.18 (−0.42, 0.07) | .16 | 9 | −0.16 (−0.32, 0.01) | .07 | .89 |
| Data visualization | 6 | −0.18 (−0.51, 0.15) | .28 | 8 | −0.17 (−0.31, −0.04) | .01 | .97 |

^a^ Subgroup analysis was not performed for the feature because there were fewer than two trials in one of the subgroups.
